# A sustainability trilogy approach for drought risk prevention: Case study in Indonesia

**DOI:** 10.4102/jamba.v17i1.1811

**Published:** 2025-06-04

**Authors:** Ramli Akhmad, Sumarmi Sumarmi, I. Komang Astina, Satti Wagistina

**Affiliations:** 1 Department of Geography, Faculty of Social Sciences, Universitas Negeri Malang, Malang, Jawa Timur, Indonesia; 2 Department of Geography, Faculty of Social Sciences and Economics, Universitas Hamzanwadi, Lombok Timur, Indonesia

**Keywords:** case study, climate change adaptation, disaster risks, drought prevention, land management, sustainability trilogy approach, water conservation

## Abstract

Drought presents a major challenge to agriculture and rural livelihoods, particularly in countries like Indonesia, which feature diverse topography and unpredictable rainfall patterns. This study addresses the critical need for a sustainable approach to mitigate drought risks by employing a ‘sustainability trilogy’ approach that integrates economic, social, and environmental dimensions. The research objectives are to: (1) identify potential risks and impacts of drought, (2) evaluate water management programmes and technologies to combat drought, and (3) apply the three pillars of sustainable development through the sustainability trilogy as a framework for drought disaster mitigation. A case study methodology was employed, focusing on Lombok, Indonesia, where water management systems and agricultural practices were examined. Data collection involved field observations, interviews, and focus group discussions (FGDs) with local farmers, irrigation managers, and community leaders. A thematic analysis combined with expert judgement analysis was used to evaluate the effectiveness of water conservation techniques, land management, and climate adaptation policies. The findings indicate that short-term solutions, such as optimising reservoirs and water storage systems, significantly reduce immediate drought impacts. Medium-term strategies, including community-driven water conservation efforts and sustainable land management practices, enhance resilience to drought. Long-term policies, such as climate adaptation initiatives and artificial rain technologies, are vital for ensuring sustainable water resource management in the context of climate change.

**Contribution:**

This research provides valuable insights and a practical framework for policymakers, aimed at strengthening water resilience, agriculture, and community sustainability in drought-prone regions.

## Introduction

Drought has become a severe global issue, increasingly threatening food security, water availability, and socio-ecological stability (Bolan et al. 2024). Numerous studies have examined the risks of drought in agriculture. De Silva and Kawasaki (2018) highlighted the economic vulnerability of low-income households in Sri Lanka due to drought and flood risks, while Guan et al. (2021) revealed that drought in China has a more significant impact than floods. Other studies in California have shown that recurring droughts have reduced agricultural productivity and intensified water-use conflicts among industries, agriculture, and households (Fernandez-Bou et al. 2023). In regions like sub-Saharan Africa, extended droughts have triggered a deepening food crisis affecting over 40 million people, exacerbating poverty (Aboye, Gebre-Egziabher & Kebede 2024). In Australia, the 2019–2020 drought led to significant agricultural losses, bushfires affecting 18 million hectares, and massive economic damage (Haque, Akbar & Kinnear 2024). Similarly, Indonesia also experiences the same drought challenges in those aspects: food security, water availability, and socio-ecological stability. As a region affected by the monsoon climate and the El Niño–Southern Oscillation (ENSO), areas such as West Nusa Tenggara (WNT) have experienced significant impacts. Approximately 68% of its 893,758 hectares of dryland are underutilised, particularly for the cultivation of rice and other staple crops, resulting in insufficient food production at the household level (Rosyadi, Darma& Darma 2023). However, these studies rarely examine local contexts, such as Lombok, Indonesia. This region struggles with low rainfall which leads to frequent droughts. The dense population makes the problem worse by increasing pressure on resources. Since most people rely on farming for their livelihood, the region is particularly vulnerable to water shortages (Nandini & Kusumandari 2022). Arabameri et al. (2022) and Gangopadhyay et al. (2023) suggest that unique and complex characteristics of drought-prone regions, particularly those experiencing meteorological, hydrological, and agricultural drought, require a sustainable approach that combines economic, social, and environmental dimensions to ensure effective risk reduction.

This study aims to bridge this gap by applying the Sustainability Trilogy Approach (STA), a framework adapted from the well-established concept of sustainable development (Daly 1990), which emphasises the interdependence of economic growth, environmental protection, and social equity in achieving long-term sustainability (Klarin 2018). Sustainability Trilogy Approach operationalises the sustainability paradigm by integrating the three dimensions of sustainability – economic, social, and environmental – into a trilogy approach for managing drought risk, adaptable to local and global contexts. This approach aligns with existing drought mitigation frameworks, as effective mitigation requires synergy between these three dimensions of sustainability; without it, fragmented efforts will fail to achieve sustainable outcomes (Leitner et al. 2020). This research employs a qualitative case study in drought-prone Central Lombok, using descriptive and interpretive methods to investigate the interplay between water conservation, land management, and climate adaptation (Dobler-Morales & Bocco 2021). The qualitative approach enables a deep understanding of local perceptions of drought, which are subjective and context-dependent (Van Den Berg & Mallick 2024). This study also captures the social and cultural factors that influence drought resilience, which are often overlooked in quantitative data (Bercht et al. 2024). Therefore, this research contributes to a comprehensive understanding of the adaptive approaches employed by communities to reduce drought risks.

## Literature review

### Understanding of drought disaster and drought potential

Drought is a natural phenomenon that commonly occurs during the dry season (He et al. 2021), especially in areas with low water reserves (De Brito et al. 2021). In Indonesia, according to the Meteorology, Climatology, and Geophysics Agency (BMKG) regulations, drought is considered a disaster if it persists for an extended period, typically three consecutive months or more (Siswanto et al. 2022). It threatens community activities, such as disrupts the lives, affecting agricultural productivity, water supply, and public health, leading to food insecurity and economic losses, and impacts on people’s livelihoods of the community (Farooq et al. 2022). Drought disaster is usually caused by natural, non-natural, or human factors, resulting in loss of life, environmental damage, property loss, and psychological impacts (Mulianingsih & Hardati 2022). The definition of drought can vary depending on its type. For instance, in this study, drought refers to meteorological, agricultural, and hydrological droughts, each of which is defined differently based on its duration and severity. Drought is also associated with a significant reduction in rainfall over a prolonged period. Typically lasting one season or more, it leads to water shortages that hinder the fulfilment of basic needs (Ahmad et al. 2022; Dąbrowska et al. 2023). Assessing drought potential involves evaluating an area’s vulnerability to drought conditions (Dikshit et al. 2022), meaning that certain areas are more prone to experiencing drought (Masroor et al. 2021).

#### Drought factors and classification based on impacts

Drought is influenced by several factors beyond mere lack of rainfall (Li et al. 2021):

- **Meteorological factors:** Climate conditions like El Niño extend the dry season.
- **Hydrological factors:** Reduced water flow in rivers leads to downstream droughts.
- **Agronomic factors:** Soil moisture deficiencies hinder plant growth.
- **Water infrastructure issues:** Inadequate reservoirs and dams limit water availability.
- **Law enforcement problems:** Water theft and facility damage complicate water distribution.
- **Socioeconomic factors:** Inefficient water use reduces community participation in water conservation efforts.

Drought can be classified by its causes and effects (Ndayiragije & Li 2022):

- **Meteorological drought:** The earliest indicator, caused by below-normal rainfall.
- **Hydrological drought:** A result of declining surface and groundwater levels, measured through rivers and reservoirs.
- **Agronomic drought:** Prolonged soil moisture deficiencies that inhibit plant growth.
- **Socioeconomic drought:** When commodity supply fails to meet demand due to other drought types.

#### Strategies for managing drought-prone areas

Disaster management focuses on enhancing preparedness and minimising risks through strategic planning, organisation, implementation, and oversight (Alam & Ray-Bennett 2021; Wulandari et al. 2023), adopting a multidisciplinary approach due to drought’s complex impact on community life (Miller & Pescaroli 2018). It includes both mitigation and prevention strategies (Yu & He 2022). Mitigation focuses on reducing risks after a disaster, while prevention aims to minimise risks before disaster strikes (Pascapurnama et al. 2018). Drought prevention includes water resource management and conservation (Marlina et al. 2022; Wang et al. 2022) and infrastructure development to distribute clean water. Prevention efforts also involve technologies such as the construction of reservoirs tailored to environmental conditions to promote sustainability (Marengo et al. 2022; Masruroh et al. 2023).

#### Integrating vulnerability factors in drought assessment

Drought disaster management requires a multidimensional assessment that integrates economic, social, environmental, and geographic perspectives (Sahani et al. 2019). One key approach is vulnerability analysis, which examines various physical and hydrological factors, including soil characteristics, rainfall patterns, landform features, channel density, and groundwater conditions (Berhanu & Hatiye 2020). Soil properties – such as type, texture, and permeability – affect water retention and infiltration rates, influencing drought severity (Ben Zaied, Jomaa & Ouessar 2021). Rainfall variability, measured through monthly and annual intensities, determines water availability and the frequency of drought occurrences. Additionally, landform analysis provides insights into the physical terrain, which influences surface runoff and water storage capacity, while channel density helps assess watershed hydrodynamics and water flow distribution (Bin Ghomash, Caviedes-Voullieme & Hinz 2019). Lastly, groundwater data, including aquifer depth and distribution, is crucial for understanding long-term water reserves and the potential for drought resilience (Seyoum et al. 2019).

### Sustainable development concept

**FIGURE 1:**
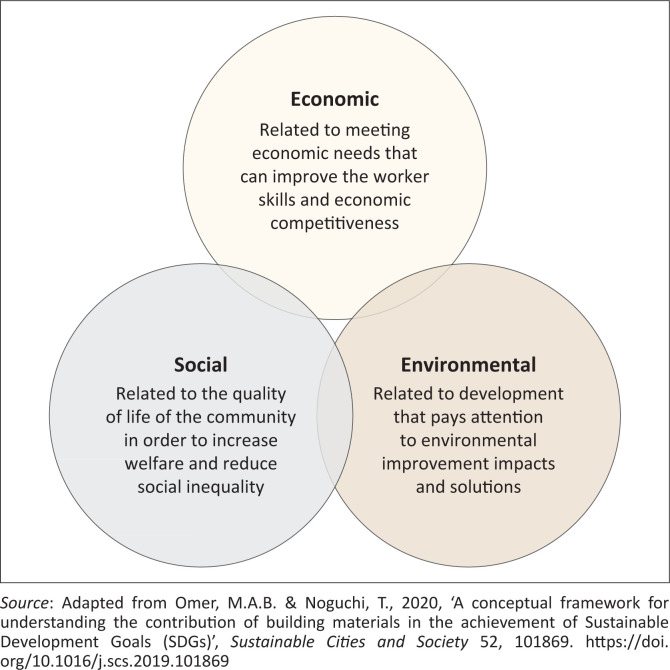
Framework for the concept of sustainable development.

Jonathan Metzger (2013), in *Sustainable Stockholm*, explains that sustainable development traces back to the Stockholm Conference in Sweden in 1972. Dissatisfaction with environmental management led to the establishment of the World Commission for Environment and Development (WCED) during a United Nations (UN) Environment Programme session in Nairobi, Kenya. Subsequent key conferences took place in Rio de Janeiro in 1992 and Johannesburg in 2002 (John 2025). Sustainable development, as defined by conservationists, promotes economic growth while preserving resources for future local generations (Payne 2006; Sumarmi, Bahcri & Tanjung 2019). More broadly, it involves meeting present needs without compromising the environment (Klarin 2018), protecting ecosystem functions, and ensuring equitable opportunities for all (Holden, Linnerud & Banister 2014). More details are illustrated in Figure 1.

According to the triangular concept of sustainable development, an activity – such as agriculture and agribusiness – is considered sustainable if it integrates economic, environmental, and social aspects (Desiderio et al. 2022). From an economic perspective, sustainable development should foster growth, conserve capital, and ensure the efficient use of resources and investments (Ye et al. 2022). Key indicators of economic sustainability include efficiency, competitiveness, value-added growth, and economic stability.

The environmental dimension emphasises the importance of preserving ecosystems, maintaining environmental carrying capacity, and conserving natural resources, including biodiversity. The focus is not on maintaining static environmental conditions but rather on enabling ecosystems to adapt to changes (Musasa & Marambanyika 2020). Social sustainability aims to ensure equitable development, promote social mobility, and strengthen institutions. This dimension encompasses poverty reduction, fair income distribution, political participation, and social stability (Hackl 2018). These three pillars – economic, environmental, and social – are interdependent and must be balanced to achieve long-term sustainability.

## Research methods and design

### Research design

This study employs a qualitative case study design using the sustainable development trilogy approach to examine how economic, social, and environmental dimensions shape drought risk management in Lombok Tengah. This approach provides an in-depth exploration of local perceptions, adaptive strategies, and institutional frameworks that influence sustainability in drought-prone areas. By analysing narratives, policies, and practices, the study uncovers complex interdependencies that inform more context-specific and holistic mitigation efforts (Christofi et al. 2021; Ciampi et al. 2022). Data collection methods include direct observation, in-depth interviews, document analysis, and focus group discussions (FGDs) centred on the economic impacts of drought, social adaptation strategies, and environmental water conservation practices. The document analysis includes government policies on drought management, local regulations (*awiq-awiq*), agricultural reports, and community action plans, which provide contextual insights into institutional responses and long-term adaptation strategies. By involving various stakeholders and combining multiple data sources, such as interviews, direct observation, and document analysis, this approach ensures data triangulation, which enhances the validity and reliability of the findings. The integration of these different data sets, not just interviews from different stakeholders, provides a more comprehensive and well-rounded perspective on the drought risk management strategies (Kayesa & Shung-King 2021).

### Participant selection and recruitment process

The research team comprised three experts: one in disaster risk management, one in environmental science, and one in social sciences with deep expertise in drought and agricultural issues. Data were collected from informants, including farmer groups and community organisations. The details of the informants are shown in Table 1.

**TABLE 1:** Relevant parties who became interview target informants.

Informants	Code	Consist of
Farmers group	Im	Irrigation managers
	Fw	Farm workers
	Lf	Landowner farmers
	Hf	Heads of farmers group
Community group	Rf	Religious figure
	Tf	Traditional figure
	Yf	Youth figure
	Cf	Community figure

Participants were recruited both face-to-face and via telephone using purposive sampling to ensure they had relevant knowledge and experience with drought (Asare-Nuamah et al. 2022). Farmer data from local governments helped identify individuals involved in water management and agriculture. The inclusion criteria focused on those directly affected by drought, such as farmers, water resource managers, agricultural experts, and government officials (Hawkins et al. 2022).

### Data collection

**FIGURE 2:**
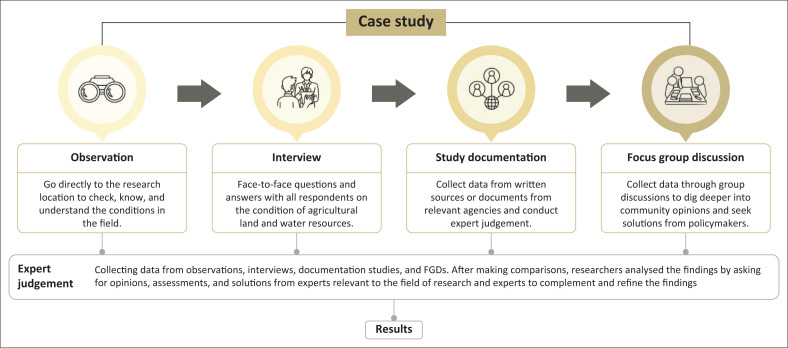
Process flow, step by step, in this research.

Data collection for this study was conducted over a period of approximately 3 months, from October to December 2023. Interviews, which lasted between 75 and 110 min (with an average of 90 min), were the primary data collection method. This was followed by field observations, document studies, and FGDs. The inclusion of FGDs in the data collection process allowed for a more dynamic and flexible approach, developing according to on initial findings to further explore issues that emerged during the study (Bwambale et al. 2022). Figure 2 shows the different methods utilised to collect data in this study.

These data collection stages were chosen to ensure comprehensive and in-depth data coverage, as each method provides complementary perspectives (Wagenaar et al. 2022). Reflectivity was carefully managed through the documentation of the researchers’ thoughts and experiences, which helped identify and reduce potential bias (Zare et al. 2021). The interview questions and FGDs were designed to be open-ended, encouraging active participation and in-depth exploration from participants. Table 2 presents information regarding the list of questions for participants from various backgrounds involved in the FGDs in this study.

**TABLE 2:** Interview questions for different participants in focus group discussions.

Participants involved in the FGDs	Interview questions in FGD
Irrigation managers	What irrigation management strategies do you implement to ensure the sustainability of water supply during the prolonged dry season?
Farm workers	Can you describe the challenges of drought that you faced as a farm worker during the dry season, and how did you overcome them?
Landowner farmers	How do you adapt cultivation techniques to maintain land productivity during the dry season?
Heads of farmers groups	What is the role of farmer groups in helping their members deal with drought problems, and what are the collective efforts made?
Religious figure	What is the role of religion in motivating communities to work together in dealing with drought disasters?
Traditional figure	Is there any local wisdom or tradition applied by the community in managing natural resources during the dry season?
Youth figure	What is the young generation’s view of the drought problem and what contribution can they make to sustainable solutions?
Community figure	What is the role of communities in dealing with drought challenges, and what are some community-based initiatives that are already running?

FGD, focus group discussion.

The questions in the FGD were formulated based on the research objective of identifying and exploring the economic, social, and environmental aspects of drought risk management in Central Lombok. Each participant group was selected based on their roles and contributions that are relevant to these three dimensions. Furthermore, the recording process was conducted using an audio recording device, while detailed field notes were meticulously documented to ensure accuracy. The data was transcribed immediately after each session to maintain the integrity of the information and enable timely analysis (Ahluwalia et al. 2022).

### Data analysis

The data analysis for this study employed thematic analysis combined with expert judgement analysis. The process began with the systematic coding of interview transcripts, field notes, and documents to identify key themes. Most coding categories were developed inductively from field findings, while some were predetermined (Batra 2021), based on the sustainable development trilogy theory, which integrates economic, social, and environmental dimensions as key pillars of sustainability (Ragheb, Aly & Ahmed 2022; Russo 2008). To ensure validity, experts from the River Region Centre, BPBD, the Agriculture Office, and BMKG were involved in the expert judgement analysis. The expert data used in this analysis is presented in Table 3.

**TABLE 3:** The experts selected to analyse the results in this research.

Experts	Field of expertise	Agency
Secretary of BWS NT 1	Management of Water Resources	River Regional Centre (BWS) NT 1
Head of BPBD	Disaster Mitigation	BPBD NTB
Head of Agriculture Office	Land and Agricultural Systems	Central Lombok Agriculture Office
Secretary of BMKG	Drought and Rainfall	BMKG NTB

BWS, Balai Wilayah Sungai; BPBD, Badan Penanggulangan Bencana Daerah; BMKG, Badan Meteorologi, Klimatologi, dan Geofisika; NTB, Nusa Tenggara Barat.

This approach strengthens the analysis by combining empirical findings with technical insights, ensuring that the recommendations are both relevant and practical (Hirose & Creswell 2023). This integration provides a comprehensive understanding of drought adaptation strategies by merging field data with expert knowledge (Naulleau et al. 2021; Singletary & Sterle 2020).

### Ethical considerations

Ethical clearance to conduct this study was obtained from the Universitas Negeri Malang Research Ethics Committee (No. 1.10.6/UN32.14/PB/2024).

This paper is part of a research involving some local community participation. Transportation compensation was provided, adhering to ethical guidelines (Fessler, Haustein & Thorhauge 2024). The participants’ rights and privacy were respected. The study’s purpose and the focus on community-based drought solutions were clearly explained to all participants (Hennink & Kaiser 2022; Laumann 2020). The participants were assured that all data provided would be kept confidential and used solely for research purposes, with their identities remaining anonymous. In Indonesia, they are considered adults; therefore, no permission from parents or legal guardians is required. The research instruments have been reviewed by the research ethics committee.

## Results

### Land characteristics and drought potential in NTB province

**FIGURE 3:**
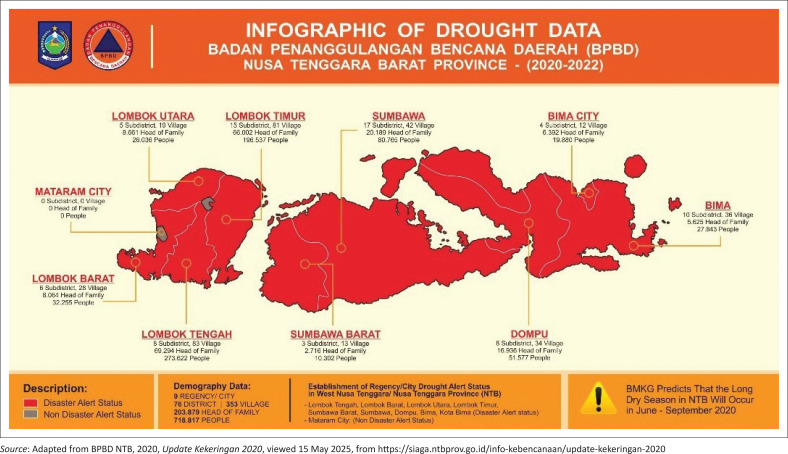
Infographic of drought data from National Agency for Disaster Counter measure NTB province.

Based on the validity of the findings through the data triangulation process, this study integrates documentation analysis with drought data from the Regional Disaster Management Agency (BPBD) of West Nusa Tenggara Province. Since August 2020, nine districts in West Nusa Tenggara (NTB) have been placed under an official drought emergency preparedness status by the regional government. This designation is based on the severity of drought conditions and the potential risks to affected communities. Among these districts, approximately 76 sub-districts, 353 villages, 203,879 households, and 718,817 people are currently identified as being in a state of emergency preparedness for drought disasters, as illustrated in Figure 3.

In an interview with Head of BPBD NTB (2023), it was revealed that nearly all regions in NTB have experienced drought over the past 3 years. In response, BPBD, in collaboration with local governments and the private sector, has been distributing clean water to affected areas. Temporary efforts by BPBD and BMKG include coordination meetings and data collection on drought-affected residents. Some regions have already begun distributing clean water, and early warnings for meteorological drought have been issued in areas that have gone over 60 days without rainfall, particularly in Dompu, Bima, Sumbawa, East Lombok, and Central Lombok.

A weighted overlay field analysis identified drought vulnerability in Central Lombok. The results indicate that 124.26 km^2^ of land has low vulnerability, 697.20 km^2^ has moderate vulnerability, and 171.19 km^2^ is classified as highly vulnerable. The region is predominantly characterised by moderate vulnerability, underscoring the high drought risk faced by Central Lombok.

Table 4 provides an overview of the extent of drought-prone areas in Central Lombok based on secondary data. To gain deeper insights into the underlying factors influencing drought vulnerability, this study integrates qualitative analysis through interviews and FGDs with key stakeholders. These high-risk zones are often situated on slopes or higher elevations, such as mountain ridges and hills, where groundwater reserves are low due to high soil permeability, allowing water to drain away quickly. In contrast, areas with low drought vulnerability, such as Janapria and Central Praya in East Praya, benefit from proximity to Mount Rinjani, which enhances water storage and results in shallower groundwater levels. Field observations suggest that drought in Central Lombok is more strongly influenced by hydrological factors, such as inadequate groundwater reserves, rather than by meteorological factors like low rainfall.

**TABLE 4:** Table of extent of drought-prone areas in Lombok Tengah regency.

Vulnerability level	Wide (km^2^)	%
Low vulnerability	124 26	1252
Moderate susceptibility level	697 20	7024
High vulnerability level	171 19	1725

**Total**	**992 65**	**100 00**

### Location and geographical conditions of agricultural land and rainfall

Central Lombok Regency is located between West Lombok and East Lombok Regencies, with coordinates of 82° 7’–8° 30’ South Latitude and 116° 10’–116° 30’ East Longitude. The total area of this regency is 1,208.39 square kilometres (120,839 hectares). Administratively, Central Lombok consists of 12 sub-districts, ranging in size from 50 to 233 square kilometres, along with 12 urban villages and 127 rural villages. Pujut sub-district is the largest, covering 233.55 square kilometres, or 19.33% of the total area of Central Lombok Regency.

**FIGURE 4:**
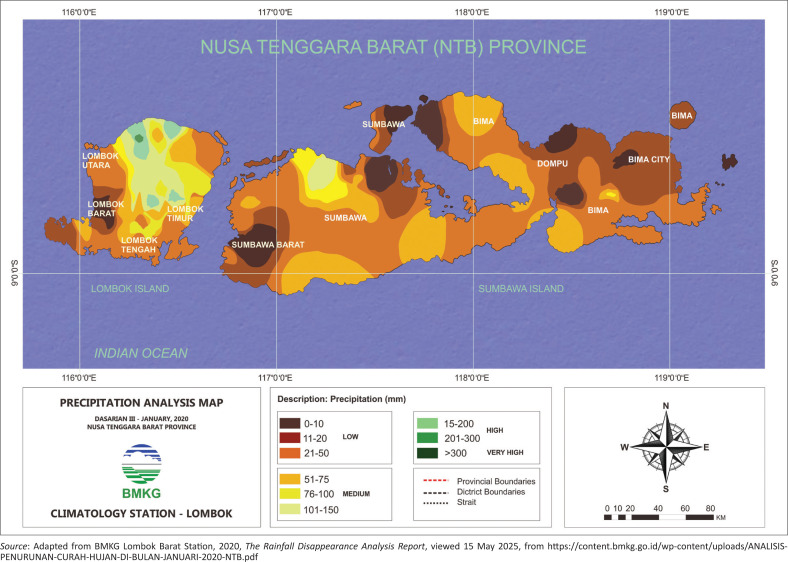
Map of average rainfall per millimetre of West Nusa Tenggara province.

Figure 4 illustrates that Central Lombok is one of the regencies in West Nusa Tenggara Province characterised by extensive dry land and low rainfall intensity. During the dry season, typically spanning from April to September, the average rainfall drops below 10 mm. In contrast, the rainy season, which lasts from October to March, sees significantly higher rainfall levels, averaging over 100 mm. January experiences the highest rainfall, reaching 448.3 mm, whereas October has the lowest, with only 0.4 mm of rainfall.

**FIGURE 5:**
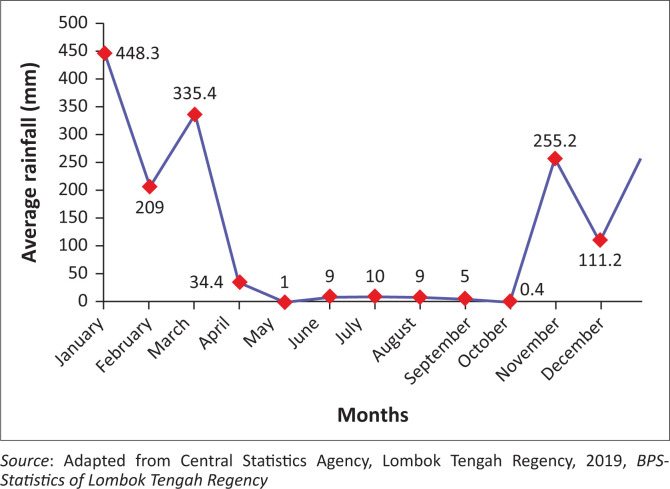
Average monthly rainfall in Central Lombok Regency, 2018.

The average monthly rainfall in Central Lombok Regency (see Figure 5) is highest in January (approximately 448.3 mm), followed by March (335.4 mm) and November (255.2 mm). A significant decline occurs between May and October, with the lowest rainfall recorded in October (0.4 mm). The dry season is clearly observed from May to October, with rainfall consistently below 10 mm per month. Conversely, the wet season spans from November to April, marked by substantially higher precipitation levels.

Data from BMKG NTB and considering altitude, soil types, and farming systems, drought vulnerability in Central Lombok is categorised into three levels: very vulnerable, vulnerable, and not vulnerable. Central Praya is classified as very vulnerable due to its low rainfall intensity of less than 60 mm per year. Six districts, including Batukliang, Pujut, East Praya, Janapria, Kopang, and Jonggat, fall into the vulnerable category with rainfall ranging between 50 mm – 100 mm per year. Five districts, namely West Praya, Southwest Praya, Praya, Pringgarata, and North Batukeliang, are classified in the high category, with rainfall between 100 mm – 200 mm per year. According to interviews with local farmers and community leaders, ‘the prolonged low rainfall has led to decreased agricultural productivity, forcing farmers to shift planting schedules and adopt water-saving techniques’. Additionally, discussions in the FGDs revealed that ‘communities rely on traditional water management practices, such as utilizing small reservoirs (embung) and collective irrigation efforts, to cope with drought conditions’.

### Alternative drought prevention based on timeframe

Drought prevention measures are generally divided into three stages: short-term, medium-term, and long-term management. These stages are aligned with the general framework of development planning. In a FGD held with the community on December 2022, participants highlighted the importance of adopting suitable and environmentally friendly technologies to ensure sustainable drought management in Central Lombok. These efforts align with the three stages of drought management, as outlined in the FGD results, which identified the drought issues in Central Lombok across three dimensions, detailed in Table 5.

**TABLE 5:** The problems of farmers due to drought in economic, social, and environmental aspects.

Aspect	Description or problems
Economic	Agricultural activities are stagnant, harvests fail, income decreases, and permanent jobs are lost as farmers and poverty rates increase.
Social	The quality of nutrition declines, access to clean water services are disrupted, water theft and crime are high, and the rate of crime increases.
Environmental	Lack of irrigation water, shortage of clean water facilities, decreased well water debit, decreased quality and quantity of agricultural products, reduced soil quality, widespread dry land.

*Source*: Interview and FGD results, 2022

Table 5 provides an overview of the various problems caused by drought in Central Lombok, categorised by economic, social, and environmental aspects. These findings were synthesised from interviews and FGDs conducted with key stakeholders, ensuring a qualitative depth to the analysis. Based on these issues, alternative drought prevention measures were identified through FGD, organised into three time frames: short-term, medium-term, and long-term. The details are explained in the following sections.

#### Short-term drought prevention

Interviews and FGDs revealed several challenges faced by the community, including a shortage of clean water for household needs, economic hardships for low-income families whose farming activities are affected by drought, outbreaks of diseases like diarrhoea, measles, pneumonia, and a decline in nutrition for toddlers in drought-prone areas. To address these problems, several prevention measures were proposed based on discussions in interviews and FGDs with key stakeholders, including village government representatives, farmer groups, and the local health department. These measures include fulfilling the immediate need for clean water through distribution facilitated by the village government, providing food assistance to low-income families affected by drought, and collaborating with the local health department to manage disease outbreaks. Based on discussions with local community leaders, concerns emerged regarding the nutritional needs of toddlers in drought-prone areas. One participant stated, ‘It is crucial to strengthen the existing nutritional support programs to ensure children receive proper nutrition during prolonged droughts’. Meanwhile, a youth representative highlighted, ‘Health care support and adequate emergency funds are essential to sustain the community during critical situations, as many families struggle to meet their basic needs’.

The average monthly rainfall in Central Lombok Regency (see Figure 5) is highest in January (approximately 448.3 mm), followed by March (335.4 mm) and November (255.2 mm). A significant decline occurs between May and October, with the lowest rainfall recorded in October (0.4 mm). The dry season is clearly observed from May to October, with rainfall consistently below 10 mm per month. Conversely, the wet season spans from November to April, marked by substantially higher precipitation levels.

#### Medium-term drought prevention

In the medium-term, problems related to water sources and inadequate clean water facilities during the dry season were highlighted. To address these issues, the following solutions were proposed by community leaders, local government officials, and farmer groups during FGDs: improving the availability of water sources, digging wells, installing hand pumps and deep wells, implementing rainwater harvesting systems, constructing water terminals in drought-prone villages, and building reservoirs. Additionally, during the FGD, local government officials, community leaders, and water resource managers recommended the importance of conducting research to explore potential water sources and improve the quality of clean water facilities.

#### Long-term drought prevention

In the long term, the community faces issues such as declining irrigation water, environmental degradation around water sources, and damage to agricultural areas due to deforestation. Long-term prevention measures include reforestation around water sources, rehabilitating green belts, land conservation, managing community forests, and constructing infiltration wells in drought-prone areas.

## Key elements of a drought prevention strategy

Drought is a natural phenomenon that occurs when there is an extended period of below-average precipitation, leading to significant economic, social, and environmental impacts. As droughts increase in frequency and severity due to climate change, sustainable approaches are necessary. The **expert judgement analysis** identified three essential elements for a drought prevention strategy:

### Water conservation

Sustainable water management, including reducing demand, improving efficiency, and managing supply systems. Water-saving technologies such as rainwater harvesting, drip irrigation, and drought-resistant crops are crucial. Public awareness campaigns should promote behaviour changes towards water conservation.

### Land management

Sustainable land use practices, such as afforestation, reforestation, and conservation agriculture, can help reduce soil erosion and increase soil moisture retention. These practices promote ecosystem resilience and mitigate drought impacts on agriculture and biodiversity.

### Climate change adaptation

Strategies to minimise the impacts of climate change on water resources and agriculture, such as developing early warning systems, promoting drought-tolerant crops, education, and creating alternative livelihoods for affected communities.

## Application of economic, social, and environmental dimensions in agricultural sustainability

Drought affects agricultural lands, causing water shortages, but sustainable agriculture can mitigate drought vulnerability by addressing three key dimensions:

### Economic sustainability

Agriculture impacts food prices, public finances, and food security. Sustainable economic strategies must consider both short-term investments, such as reservoirs and irrigation systems, and long-term macroeconomic implications.

### Social sustainability

Agriculture influences food variety and community vitality. Urbanisation and societal changes can affect farmland availability, and sustainable agriculture must balance these dynamics to ensure access to nutritious, affordable food.

### Environmental sustainability

Agricultural practices can cause soil erosion and water depletion. A holistic approach to environmental sustainability requires integrating social and economic factors with long-term ecological management.

## Discussion

The research highlights that, given the region’s diverse topography and uneven rainfall, sustainability solutions must be integrated across short, medium, and long-term strategies. The **Trilogy of Sustainability Approach** is crucial for ensuring water security and effectively mitigating drought risks in the Lombok region of Indonesia. This approach synergises technology, policy, and community participation, incorporating the three main dimensions of sustainable development: economic, social, and environmental (Giannetti et al. 2019; Klarin 2018).

From an **economic perspective**, water management strategies should consider long-term economic sustainability. Short-term solutions, such as constructing and optimising reservoirs and dams, provide direct economic benefits by enhancing water security in drought-prone areas (Desiderio et al. 2022). For example, revitalising the Batu Jai reservoir in Central Lombok and Pandandure Dam in East Lombok has successfully increased water storage capacity, supporting the local agricultural economy (Hamdan et al. 2022). Medium-term efforts, such as water conservation technologies, help optimise agricultural yields while reducing costs, maintaining the competitiveness of the agricultural sector (Ye et al. 2022).

In the **social dimension**, community engagement is essential to drought risk mitigation. Medium-term public awareness programmes focusing on water conservation will foster community participation in drought-prone areas (Paez-Trujillo et al. 2024). Long-term climate change adaptation strategies require social support, especially for innovations like artificial rain. Although controversial, such solutions can be effective if properly evaluated for environmental impact (Liu et al. 2021).

In terms of the **environment**, maintaining ecosystem balance and preserving natural resources are critical components of drought mitigation. Long-term strategies should focus on conserving biodiversity through sustainable land management policies (Savari, Damaneh & Damaneh 2023). Water conservation technologies, including artificial rain, must be thoroughly assessed for long-term environmental impact to ensure that they do not harm ecosystems. Integrating technology, education, policy, and community involvement will foster ecological resilience in regions vulnerable to climate change (Prasad 2022).

Ultimately, the Sustainability Trilogy Approach, combining economic, social, and environmental aspects, demands close collaboration between technology, government policies, and community groups. By adopting this model, Lombok and other drought-prone areas in Indonesia and beyond can effectively reduce drought risk, strengthen resilience to climate change, and support sustainable development (Klarin 2018; Rana 2020).

## Conclusion

The **Sustainability Trilogy Approach** to drought risk prevention in Lombok, Indonesia, underscores the vital integration of economic, social, and environmental dimensions. Drought-prone areas can enhance water security and agricultural sustainability through water management strategies that are adapted to local characteristics. Implementing measures over three time horizons – short, medium, and long term – requires the synergy of technology, government policies, and community engagement to achieve sustainable outcomes. Drought risk can be substantially mitigated through social engagement, economic efficiency, and ecosystem preservation.

This study reveals key findings that provide valuable insights for policy and practice. Empirical data from interviews and FGDs indicate that policymakers need to strengthen technology-based water management policies tailored to local climatic conditions. Participants emphasised the importance of sustainable investment in water infrastructure, particularly in the construction of reservoirs and the optimisation of existing irrigation systems to enhance drought resilience. Additionally, community involvement in water conservation emerged as a central theme. Local communities and farmers proposed greater engagement from academics in expanding educational programmes and raising public awareness to protect water resources while strengthening adaptive capacity. Furthermore, discussions highlighted concerns about the impacts of drought, underscoring the need for comprehensive climate adaptation policies that balance innovation with social, economic, and environmental sustainability. The Sustainability Trilogy Approach, as validated through this study, offers a practical framework not only for Central Lombok but also for other drought-prone regions, integrating social, economic, and environmental dimensions to enhance long-term resilience to climate change.
